# Evaluation of cell types for assessment of cytogenetic damage in arsenic exposed population

**DOI:** 10.1186/1476-4598-7-45

**Published:** 2008-05-28

**Authors:** Pritha Ghosh, Arindam Basu, Keshav K Singh, Ashok K Giri

**Affiliations:** 1 Molecular and Human Genetics Division, Indian Institute of Chemical Biology, Kolkata-700 032, India; 2 Department of Cancer Genetics, Roswell Park Cancer Institute, Buffalo, New York 14263, USA

## Abstract

**Background:**

Cytogenetic biomarkers are essential for assessing environmental exposure, and reflect adverse human health effects such as cellular damage. Arsenic is a potential clastogen and aneugen. In general, the majority of the studies on clastogenic effects of arsenic are based on frequency of micronuclei (MN) study in peripheral lymphocytes, urothelial and oral epithelial cells. To find out the most suitable cell type, here, we compared cytogenetic damage through MN assay in (a) various populations exposed to arsenic through drinking water retrieved from literature review, as also (b) arsenic-induced Bowen's patients from our own survey.

**Results:**

For literature review, we have searched the Pubmed database for English language journal articles using the following keywords: "arsenic", "micronuclei", "drinking water", and "human" in various combinations. We have selected 13 studies consistent with our inclusion criteria that measured micronuclei in either one or more of the above-mentioned three cell types, in human samples. Compared to urothelial and buccal mucosa cells, the median effect sizes measured by the difference between people with exposed and unexposed, lymphocyte based MN counts were found to be stronger. This general pattern pooled from 10 studies was consistent with our own set of three earlier studies. MN counts were also found to be stronger for lymphocytes even in arsenic-induced Bowen's patients (cases) compared to control individuals having arsenic-induced non-cancerous skin lesions.

**Conclusion:**

Overall, it can be concluded that MN in lymphocytes may be superior to other epithelial cells for studying arsenic-induced cytogenetic damage.

## Background

Exposure to inorganic arsenic through drinking water results in cancers of skin, urinary bladder, liver and the lungs. Cytogenetic assays play an important role in toxicological hazard evaluation as the first step towards quantification of cancers. Various genetic toxicological end-points have been used as biomarkers to understand the biological effects of arsenic exposure [1]. Biomarkers serve as internal indicators of environmental or occupational exposures and have the potential for prevention of effects of carcinogen exposure by early detection [2]. Micronuclei are the potential biomarkers and traditionally been used for biomonitoring of genotoxic effects in humans [3]. Micronuclei are chromosomal fragments or the whole chromosomes that are not included in to the daughter nuclei during cell division and are incorporated as much smaller nuclei. The formation of MN is therefore induced by substances that cause breakage of chromosomes (clastogens) as well as by agents, which affect the spindle apparatus (aneugens). The speed and ease of MN analysis, and non-requirement of metaphasic cells made this assay very popular.

In West Bengal, India, the ground water in nine out of eighteen districts is heavily contaminated with arsenic. More than 6 million people are endemically exposed to inorganic arsenic in their ground water, which exceed the maximum contamination level of 10 μg/l [4,5]. Although only 15–20% of the population exposed to arsenic shows clinical features of chronic arsenic poisoning, the remaining individuals are also at risk of developing arsenic-induced health effects [6]. Therefore, genetic monitoring of population exposed to arsenic through drinking water is important. Numerous population-monitoring studies have been carried out in various arseniasis endemic countries such as Mexico, Finland, Taiwan and Argentina to determine genotoxic potential of arsenic [1,7-9].

MN assay are conducted in lymphocytes, urothelial, and buccal mucosal cells. Cytokinesis-block micronucleus test (CBMN) in human peripheral blood lymphocytes is a standard cytogenetic test for genetic toxicology testing [10]. The use of exfoliated cells for MN assays has become well established in epidemiological studies aimed at defining genotoxic effects on target tissue following chronic exposure to epithelial carcinogens [2]. We have been working on the mutagenic and genotoxic effects of different drugs, chemicals and environmental toxicants, including arsenic [11-16]. Last five years we have been working on the assessment of cytogenetic damage as measured by MN assays in three cell types i.e. lymphocytes, urothelial cells and buccal mucosa cells in the population exposed to arsenic through drinking water in West Bengal, India.

Peripheral lymphocytes can be used as surrogate target cells [17]. Collection of lymphocyte is socially and ethically acceptable, and involve minimum invasive route. The cytokinesis-block micronucleus (CBMN) technique in lymphocyte culture is widely regarded as a sensitive and reliable method for assessing chromosome damage [18]. It is based on the use of cytochalasin B, an inhibitor of actin polymerization, which blocks mitotic cytokinesis without preventing nuclear division. In this assay MN are scored after a single cell division using binucleated lymphocytes to eliminate the confounding effect of altered cell division kinetics on the MN index [18].

Exfoliated epithelial cells have traditionally been used for cancer screening and bio-monitoring of genotoxic effects in human [3]. Large number of these cells can be rapidly and non-invasively collected from study participants. The frequencies of MN observed in exfoliated cells of buccal mucosa and urinary bladder serve as an appropriate index to monitor the genotoxicity induced by arsenic because these cells are in direct contact with the carcinogen [2]. MN in urothelial cells reflects damage to the bladder epithelial tissue, which occurs approximately 1–3 weeks prior to the exfoliated cells appearing in urine [19].

Although all three-cell types provide important information about cytogenetic damage, selection of an appropriate cell type is important. On one hand, life span of lymphocytes is higher than that of the other two exfoliated cell types, and on the other hand, turn over of exfoliated epithelial cells is variable, therefore, it is reasonable to assume that lymphocytes might yield higher MN count/1000 cells compared to other exfoliated cells.

We earlier reported MN counts in three different cell types from the same individual. In this paper, we extend the research based on our systematic literature review and comparing the summary data with findings from our earlier research. We have also conducted a matched case-control study taking arsenic-induced Bowen's patients as cases and age-sex matched individuals with arsenic-induced non-cancerous skin lesion as control. The purpose is to study (a) which cell type show stronger effects on arsenic exposure and (b) whether this cell type is also similarly informative for cancer patients.

### Search strategy & inclusion/exclusion criteria

We searched the Pubmed literature database using the following keywords in various combinations: "arsenic", "micronuclei", "drinking water", and "human" [20]. We restricted our selection of studies to those published in English language only since we did not have facilities for translations nor did we have access to translation services. Initially, titles and abstracts were scanned to identify whether the study met the minimum inclusion criteria to be retained for review. We selected only case-control studies. If the study met the minimum criteria, then full text of the study was obtained. Data were abstracted from each study to a pre-defined database and the database was prepared for analysis. The reference section of each study was scanned to identify other similar articles.

A study was included in our review if it contained the following information as minimum: clearly written methods section, unbiased or minimally biased measurement of arsenic exposure, micronuclei counts per 1000 binucleated cells either in one or more of the three cell types, such as- lymphocyte, urothelial and buccal mucosa cells, and clear presentation of the results in the format of difference in effect sizes of MN per 1000 cells. Where the counts were not reported per 1000 binucleated cells, if the study met the other criteria and was accepted, we converted the figures to reflect changes per 1000 cells and rounded off the decimal points to two places after zero. Studies with non-human samples were also excluded.

### Analysis of literature review

Our literature search retrieved initially a list of 20 publications. After filtering through inclusion and exclusion criteria, we found 13 studies to be eligible (Table 1).

**Table 1:** Micronuclei in three different cell types for all the studies included in the review

			Effect size [Exposed; vs. Unexposed; Mean (SE)]		
			
Sl no.	Author, Year [Ref No.]	Sample Size N (Exposed; Unexposed)	Lymphocyte	Urothelial	Buccal
1	Warner et al., 1994. [23]	36 (18,18)	-	2.79 (0.73) vs. 1.57 (0.28)	-
2	Moore et al., 1996 [24]	36 (18,18)	-	2.8 (NA) vs. 1.67 (NA)	-
3	Dolout et al., 1996 [9]	44 (22; 22)	38 (3.2) vs 6.9 (1.7)		
4	Moore et al., 1997 [25]	125 (70; 55)	-	3.2 (NA) vs. 2.6 (NA)	-
5	Biggs et al., 1997 [26]	104 (83; 21)	-	3.34 (NA) vs. 1.61 (NA)	-
6	Gonsebatt et al., 1997 [7]	69 (35; 34)	-	2.23 (0.99) vs. 0.48 (0.10)	2.21 (0.47) vs. 0.58 (0.13)
7	Tian et al., 2001 [27]	32 (19; 13)		1.44 (0.37) vs. 0.53 (0.14)	2.21 (0.36) vs. 0.65 (0.21)
8	Basu et al., 2002 [22]	65 (45; 21)	6.39 (0.25) vs. 0.53 (0.07)	5.74 (0.27) vs. 0.56 (0.1)	5.15 (0.3) vs. 0.77 (0.11)
9	Basu et al., 2004 [15]	317 (163; 154)	9.34 (0.153) vs. 1.66 (0.061)	6.65 (0.13) vs. 1.41 (0.05)	5.94 (0.15) vs. 1.28 (0.05)
10	Martinez et al., 2004 [21]	217 (106; 111)	14.44 (0.99) vs. 11.96 (1.02)	-	-
11	Martinez et al., 2005 [28]	207 (105; 102)	-	-	3.14 (0.32) vs. 2.74 (0.26)
12	Chakraborty et al., 2006 [29]	70 (45; 25)	-	-	9.8 (0.7) vs. 2.9 (0.1)
13	Ghosh et al., 2006 [16]	306 (204; 102)	7.76 (0.17) vs. 2.03 (0.08)	5.13 (0.13) vs. 1.70 (0.07)	4.62 (0.15) vs. 1.67 (0.06)

#### Lymphocyte as a cell type for MN assay

To our knowledge, only five studies document lymphocyte as a cell type for arsenic-induced MN formation. About 5 fold increase in MN lymphocytes was observed among native children and women of northwestern Argentina exposed to high level of arsenic via drinking water in contrast to controls by Dulout et al., 1996 [9]. Higher number of MN formation in lymphocyte due to arsenic exposure was observed from the studies of Martinez et al., (2004) in the population of Northern Chile [21]. Since a large number of individuals are exposed to arsenic in West Bengal, India, three separate studies were conducted from different region of West Bengal, India and it was observed that, MN in lymphocyte is much higher in cases compared to control [15,16,22].

#### Urothelial as a cell type for MN assay

Relatively more studies were performed with urothelial cell for MN assay. Studies by Warner et al (1994) in a population exposed to arsenic in Neveda, USA showed about 1.8 fold increase in the mean frequency of micronucleated urothelial cells compared to control [23]. Using advanced technology for detecting MN by fluorescent method, Moore et al (1996) observed similar result for urothelial cells [24]. Further, they have showed higher prevalence of MN in urothelial cell in cases compared to control, in Chilean population [25]. To find out the relationship of urinary arsenic (an estimate for arsenic intake) and a biomarker of effect, prevalence of MN was found to be 2 fold higher in cases compared to control [26]; while Gonsebatt et al. (1997) have shown 4.65 fold increases [7]; and Tian et al (2001) observed about 3 fold increases in mean MN count for urothelial cells in cases [27]. All the three studies from our group strongly support increase mean MN count in urothelial cell in arsenic exposed cases compared to control [15,16,22].

#### Buccal mucosa as a cell type for MN assay

Gonsebatt et al. (1997) have shown about 3.8 fold increase in MN in buccal cells in exposed individuals compared to unexposed [7]. Similar inference was also obtained from the studies of Tian et al. [27]. MN frequency in buccal cells of arsenic exposed population from Antofagasta region, North Chile was shown to be higher compared to the referent individuals from Concepcion, Chile; but not statistically significant [28]. Again, a recent study by Chakraborty et al. (2006), on 45 arsenic exposed individuals from West Bengal revealed 3.34 fold increases in MN in buccal mucosa cells compared to 25 controls studied [29].

#### Finding best suitable cell type

From the reported data in each study, first, effect sizes were calculated as follows. Effect size was defined as the difference in the mean value of the cases versus controls. The mean value of micronuclei count per 1000 for controls were deducted from the mean value of the micronuclei per 1000 for cases, and the difference was identified as the effect size for the specific study. Effect sizes were calculated for all studies identified by the previous search strategy. All studies did not calculate the values for micronuclei counts for all three-cell types in the same individual. In that case, we treated non-reported data as missing for that study.

Since this review was not a meta-analysis, but a structured mini-review of observational studies, we did not conduct formal tests of heterogeneity or homogeneity of studies for this review. We plotted the medians of the effect sizes of each cell type as individual bar charts to show the visual differences in effect sizes among the three cell types. We reported further pairwise comparisons for the three combinations: lymphocyte versus urothelial cells, lymphocyte versus buccal cells, and urothelial cells versus buccal cells. We used the nonparametric Wilcoxon rank sum tests for the pairwise comparisons with a pre-specified significance level of 0.05. We conducted the tests for two sets of studies: (i) all studies taken together and (ii) studies that were reported from our research group.

**Figure 1:**
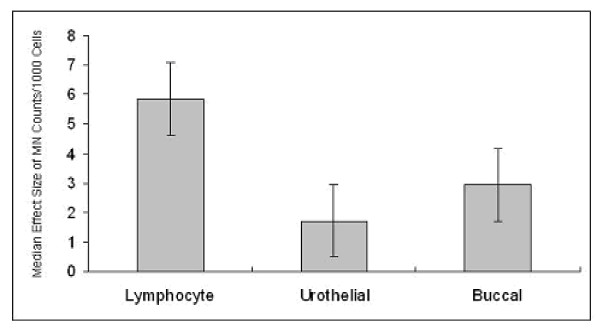
Comparison of median effect size (with error bar) based on micronuclei counts among lymphocytes, urothelial, and buccal mucosa for all the studies in the review.

**Figure 2:**
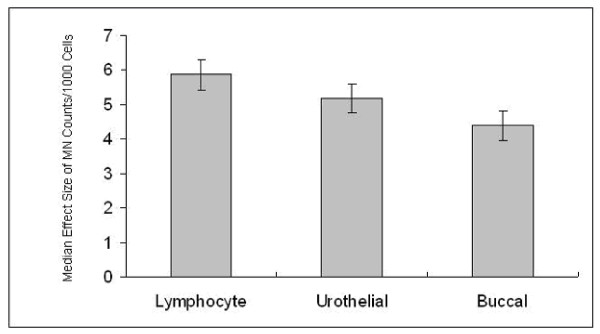
Comparison of median effect size (with error bar) based on micronuclei counts among lymphocytes, urothelial, and buccal mucosa for the subset data (set of studies from our group).

Wilcoxon rank sum test for the pairwise comparison for median effect size (based on MN counts) in three cell types for the full set of studies (Table 2) as also studies conducted by our group (Table 3) was calculated. Reviewing all the studies, it was observed that, lymphocyte is a better candidate compared to other two (Fig. 1). Similar inference was observed from the studies of our group, where MN counts were taken for all the 3 cell types for each individual. The data also supports that lymphocyte is a better cell type compared to other two (Fig. 2).

**Table 2:** Wilcoxon rank sum test for the pairwise comparison for median effect size (based on MN counts) in three cell types for the full set of studies

Median value of the effect size of MN counts^a^				
		
Lymphocytes	Urothelial cells	Buccal Mucosa cells	Comparison	Significance
5.86	1.73		Lymphocytes vs Urothelium	0.04
5.86		2.95	Lymphocytes vs Buccal cells	0.11
	1.73	2.95	Buccal cells vs Urothelium	0.48

^a ^Effect size = MN count of cases – MN count of controls.

**Table 3:** Wilcoxon rank sum test for the pairwise comparison for median effect size (based on MN counts) in three cell types for the studies conducted by our group

Median value of the effect size of MN counts^a^				
		
Lymphocytes	Urothelial cells	Buccal Mucosa cells	Comparison	Significance
5.86	5.18		Lymphocytes vs Urothelium	0.05
5.86		4.38	Lymphocytes vs Buccal cells	0.05
	5.18	4.38	Buccal cells vs Urothelium	0.10

^a ^Effect size = MN count of cases – MN count of controls.

### Is the observation also true for arsenic-induced Bowen's (in situ carcinoma) patients?

To evaluate whether the best suitable cell type suggested from literature review, also applicable in arsenic-induced Bowen's cases, we considered 25 cases of histopathologically confirmed arsenic-induced Bowen's patients from our epidemiological survey [30] and conducted a matched case-control study taking non-cancerous skin lesions individuals as control. These histologically confirmed 25 cases of Bowen's disease (designated as "case") were pair wise matched with 25 cases of non-cancerous arsenicosis individuals (designated as "control") on age, gender and smoking status (Table 4). The selection of control individuals was done via the generation of random numbers using MS-Excel spreadsheet program. Micronuclei results for all three cell types of these Bowen's cases were not reported elsewhere.

**Table 4:** Comparison of Micronuclei in three different cell types in arsenic exposed cases (Bowen's patients) and control (individuals with non- cancerous arsenic induced skin lesions)

Variable	Case (N = 25)	Control (N = 25)	Significance
*Male(N)*	*19*	*19*	Matched
*Female(N)*	*6*	*6*	Matched
Age in years	47.4	46.7	Matched
Smoker	12	12	Matched
Mean MN count in lymphocyte (SD)	11.30 (3.48)	9.10 (2.61)	< 0.01
Mean MN count in urothelial cells (SD)	7.11 (2.44)	5.80 (1.86)	< 0.05
Mean MN count in oral cells (SD)	5.85 (2.12)	4.74 (1.75)	< 0.04

Comparing the mean MN count for three cell types between cases and controls, we found MN count was significantly increased in cases for all the cell types, i.e. in lymphocyte, urothelial and oral mucosa cells (Table 4). To our surprise, MN count in lymphocyte was 1.9 fold higher compared to oral mucosa cells and 1.6 fold higher compared to urothelial cells, in both cases as well as control (Table 4).

## Conclusion

All the studies on arsenic exposed populations clearly demonstrated a significant genotoxic effect in lymphocytes as well as exfoliated epithelial cells. We have found that overall, compared to urothelial cells and buccal mucosal cells, the median effect sizes for lymphocyte based MN counts were stronger. This inference was further supported by the general pattern pooled from other 10 studies conducted by different groups. Lymphocyte based MN count was also stronger for arsenic-induced Bowen's patients. Therefore, it can be inferred that lymphocyte is the most suitable cell type for studying cytogenetic damage.

The findings from our review need to be interpreted in the light of its several limitations. We considered here only studies in English language. There may also be additional unexplained variabilities, including scoring variability among different study groups. Exposure level of the study groups also varies considerably, which might influence in outcome. It has been known from in vitro studies that, MN prevalence increase along with exposure but again return to baseline when, exposure is highest, possibly, due to inhibition of MN formation at high doses due to cytotoxicity/cytostasis [31,32].

Arsenic generates reactive oxygen species (ROS) including peroxy radical, superoxide radical, and hydroxyl radical and these in turn cause DNA damage [33]. Moreover, arsenic is a known clastogen and an aneugen, which could give rise to chromosomal mal-segregation leading to MN formation. Significant chromosomal aberrations are observed in peripheral lymphocytes when exposed to arsenic [1,7,8,16,34]. Except for a few negative results, the majority of the cytogenetic studies clearly indicated a positive clastogenic effect in population exposed to arsenic. Since, chromosomal aberration primarily lead to MN formation, lymphocyte can be used as suitable biomarker to study genotoxic effects of arsenic.

In conclusion, lymphocyte is an excellent as also the most suitable cell type for the analysis of cytogenetic damage as measured by micronuclei formation in the individuals exposed to arsenic through drinking water, and is also applicable in arsenic-induced Bowen's patients.

## Abbreviations

MN: micronuclei; CBMN: cytokinesis-block micronucleus

## Competing interests

The authors declare that they have no competing interests.

## Authors' contributions

PG and AKG were involved in designing the study and acquisition of data, AB performed the statistical analysis, KKS wrote the draft of the manuscript to which all authors subsequently contributed. All authors made contribution to the interpretation of results, and revised the manuscript for important intellectual content. All authors read and approved the final manuscript.
